# Supplementary material for: Spontaneous droplet transport on shape-evolving microfiber rails
Source: Nat Commun. 2025 Dec 4;16:10894. doi: 10.1038/s41467-025-65884-9 (PMC12678407; doi:10.1038/s41467-025-65884-9)
Supplement: Supplementary file 1 — Supplementary Information [file 41467_2025_65884_MOESM1_ESM.pdf]

Supplementary Information for

Spontaneous droplet transport on shape-evolving microfiber rails

Shiyu Wang[1], Ying Zhou[1], Wenchang Zhao[1], Yanhong Li[1], Shuxian Tang[1], Ting Si[2]*, Pingan Zhu[1,3]*

[1]Department of Mechanical Engineering, City University of Hong Kong, Hong Kong, China.

[2]Department of Modern Mechanics, University of Science and Technology of China, Hefei, China.

[3]Shenzhen Research Institute of City University of Hong Kong, Shenzhen, China.

*Corresponding author. Email: tsi@ustc.edu.cn; pingazhu@cityu.edu.hk

Supplementary Note

Supplementary Note S1: Force generated by microfiber deformation

As shown in Supplementary Fig. S6, a PDMS cantilever beam is positioned vertically against the smooth side of a horizontally straightened, dehydrated microfiber. The cantilever has a total length of l, and the distance from the fiber contact point to the fixed point is a. The contact area between the cantilever and the fiber has dimensions n (along the fiber) and m (perpendicular to the fiber). When a water droplet wets the fiber, the deformation of the hydrated fiber leads to a deflection y at the free end of the cantilever. The force exerted by the hydrated fiber can be calculated using the formula:

$F_d = 6EIy/a^2(3l - a)$,

where E is the Young's modulus of the cantilever beam, and the moment of inertia $I = nm^3/12$. Given the values $E = 1.6$ MPa, $l = 10.98$ mm, $a = 3.72$ mm, $m = 1.166$ mm, $n = 1.384$ mm, and measured deflection $y = 0.267$ mm, we find $F_d \approx 1.15$ mN. This force is an order of magnitude larger than the capillary suction force induced by a water droplet on a microfiber (with a diameter $D_m \sim 500$ μm), which is given by:

$F_s \approx \pi D_m \gamma \approx 0.113$ mN,

where $\gamma = 72$ mN/m is the surface tension of water.

Supplementary Note S2: The separation speed at the left and right sides of the droplet

When the droplet spreads to its maximum length L_s and begins to deform into a spindle-like shape due to liquid wetting, the microfiber separation speed at the center of the droplet (U_c) is the fastest, as shown in Fig. 4d. This results in a wedge-like structure on either side of the droplet. Assuming a uniform wedge with a constant wedge angle along the microfiber, we estimate that the separation speeds at the left (U_l) and right (U_r) sides of the droplet are proportional to the distances from the left and right boundaries of the SEMR, respectively (Fig. 4d). Therefore, we can express the separation speeds as:

$U_l/U_c = (L_d - L_s/2)/L_d = 1 - L_s/2L_d$,

$U_r/U_c = [L_f - L_d - L_s/2)]/(L_f - L_d) = 1 - L_s/2(L_f - L_d)$,

where L_d is the distance from the left boundary to the droplet's initial position and L_f is the total length of the microfiber. By replacing L_d with $L_d = \varepsilon L_f$, we obtain the ratio of the separation speeds at the left and right sides of the droplet as:

$U_l/U_r = (1 - L_s/2L_d)/[1 - L_s/2(L_f - L_d)] = (1 - L_s/2\varepsilon L_f)/[1 - L_s/2(1 - \varepsilon)L_f]$.

Supplementary Note S3: Laplace pressure difference across a droplet confined between two angled fibers

Consider a completely wetting fluid droplet confined between two angled fibers, each with radius R_f, forming saddle-shaped menisci at both ends of the droplet (Supplementary Fig. S9a). In the top view, the left and right edges of the droplet approximate circular arcs with radii R_l and R_r, respectively. At point A, located infinitely close to the three-phase contact line on the left edge, the Laplace pressure is determined by the Young–Laplace equation:

$$P_A = P_0 + \gamma(1/R_h + 1/R_v),$$

where P_0 is the atmospheric pressure, γ is the surface tension of the droplet, $R_h = -R_l$ is the horizontal (concave) principal radius of curvature in the top view, and $R_v = R_f$ is the vertical (conves) principal radius conforming to the fiber surface. Substituting these values yields:

$$P_A = P_0 + \gamma(1/R_f - 1/R_l).$$

Assuming quasi-static conditions (i.e., no internal flow) and negligible gravitational effects (valid when the droplet's half-height is smaller than the capillary length, approximately 2.7 mm for water), the Bernoulli principle implies that any point a on the left meniscus, coplanar with point A, has the same pressure:

$$P_a = P_A = P_0 + \gamma(1/R_f - 1/R_l).$$

Similarly, at a point b on the right edge of the droplet, the Laplace pressure is:

$$P_b = P_0 + \gamma(1/R_f - 1/R_r).$$

Therefore, the Laplace pressure difference across the droplet is:

$$\Delta P = P_a - P_b = \gamma(1/R_r - 1/R_l).$$

Experimental validation using a completely wetting fluid (ethanol, $\gamma \sim 22$ mN m^{-1}) confined between nonparallel glass fibers shows strong agreement with this theoretical prediction (Supplementary Fig. S9b and S9c), confirming the accuracy of the model.

Supplementary Note S4: Characteristic time for wicking and microfiber deformation

For the wicking process, the diffusion distance of water within the fiber can be estimated by $L \sim \sqrt{Dt}$, where D is the diffusion coefficient and t is time. From this, the characteristic time for the wicking process can be derived as $t_w \sim L_c^2/D$, where L_c represents the periodic distance between two adjacent protrusions on the microfiber.

Consider a droplet with volume Ω placed on the SEMR, where the initial spacing between the two microfibers is s_0. Over time, the droplet forms a liquid column of characteristic length L_{drop} and width W_{drop}. The droplet volume can be expressed as:

$$\Omega \sim L_{drop}^2 W_{drop}.$$

Defining the droplet's width-to-length aspect ratio as $C = W_{drop}/L_{drop}$, we can express the width of the droplet as:

$$W_{drop} \sim (\Omega C^2)^{1/3}.$$

The droplet will break up when its aspect ratio exceeds a critical value C_{criti} during the receding stage, corresponding to a critical droplet width $W_{criti} \sim (\Omega C_{criti}^2)^{1/3}$.

Consequently, the characteristic time for this process, during which the droplet reaches the critical breakup stage, is estimated as:

$$t_d \sim [(\Omega C_{criti}^2)^{1/3} - s_0]/U_c,$$

where U_c is the separation speed of the microfiber rail at the center of the droplet.

Supplementary Figures

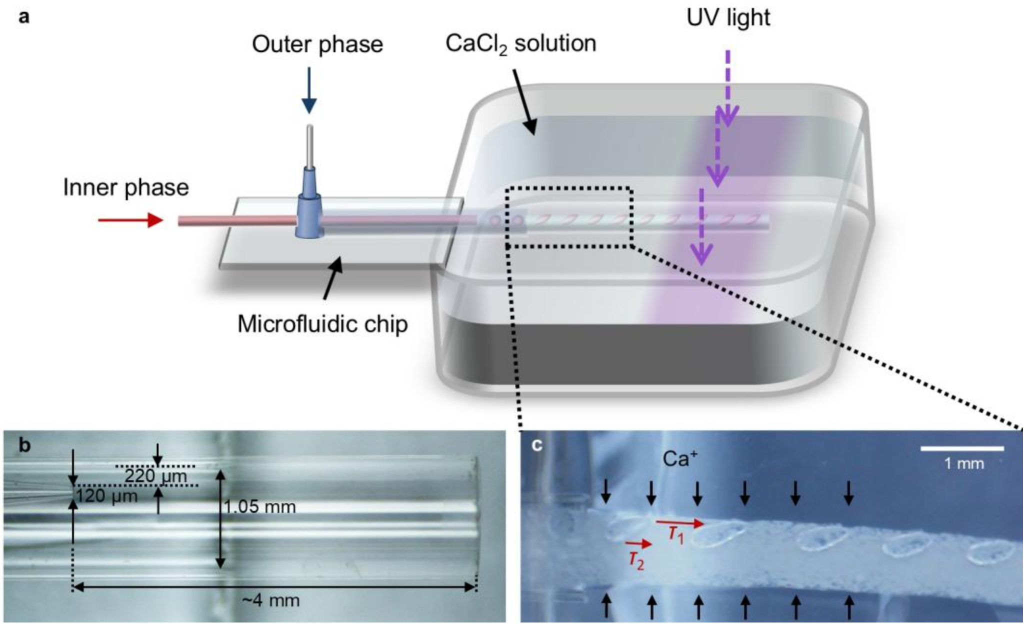

Supplementary Fig. S1: Fabrication of the microfiber for SEMR. a, Schematic of the co-flow microfluidic platform used for fabricating microfibers. **b**, Microscopic image of the microchannel configuration. An inner glass microcapillary (tip diameter of 120 μm) is eccentrically inserted into an outer microcapillary (inner diameter of 1.05 mm), with the closest distance between the inner microcapillary and the wall of the outer microcapillary being 220 μm. This eccentric microcapillary alignment enables the asymmetric distribution of microdroplets along one side of the resulting microfiber. **c**, Micrograph showing the generation of microfibers as they exit the outlet into the $CaCl_2$ solution for cross-linking. Both the shape and distribution of encapsulated microdroplets (used for templating microparticles after solidification) are asymmetric within the microfiber. τ_1 and τ_2 denote the viscous shear forces exerted on oil droplets from regions near and far from the microfiber surface, respectively, with $\tau_1 > \tau_2$.

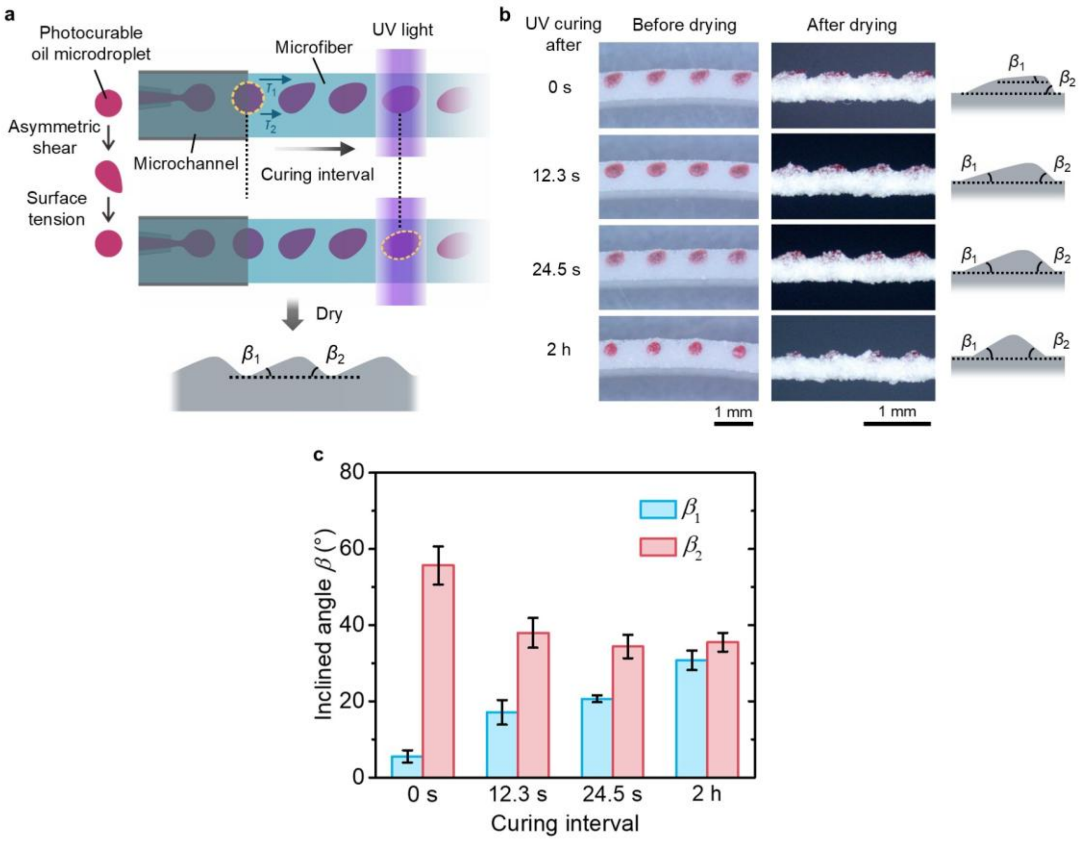

Supplementary Fig. S2: Tunable surface morphologies of microfibers. a, Tailoring microfiber morphology by adjusting microparticle shape through varying curing intervals. **b**, Comparison of microfiber morphologies before and after drying under different curing intervals. **c**, Tuning inclined angles (β_1 and β_2) achieved by controlling the curing interval. Error bars indicate the standard deviation for five measurements at each data.

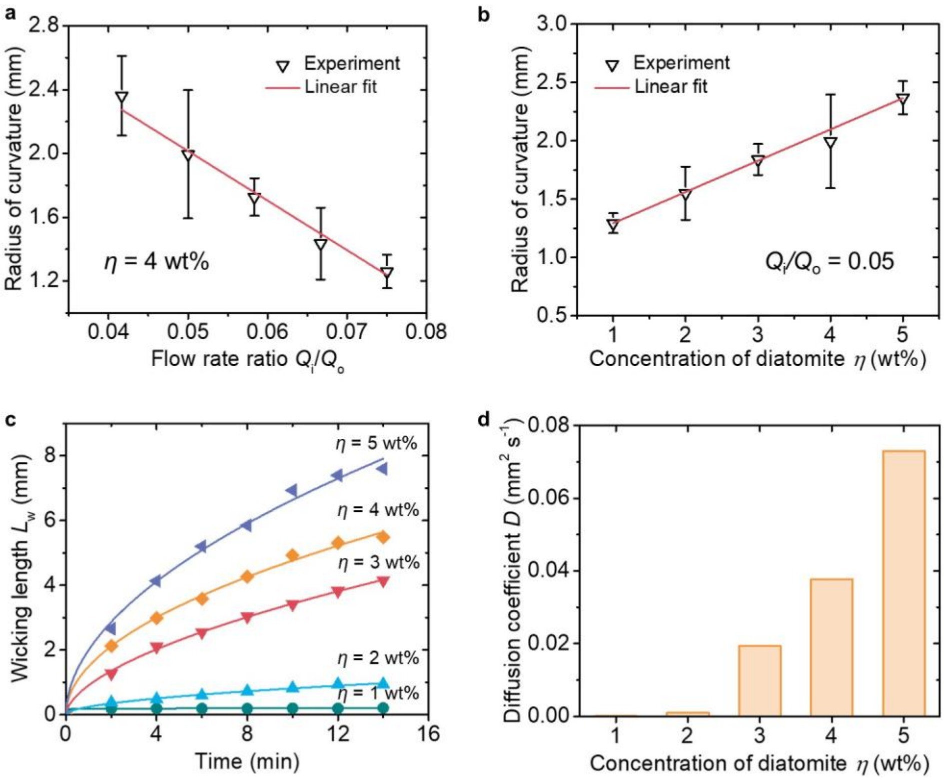

Supplementary Fig. S3: Tunable deformation and wicking features of microfibers. **a**, **b**, Control of the radius of curvature of microfiber deformation by adjusting (**a**) the flow rate ratios Q_i/Q_o and (**b**) diatomite concentration η in the alginate–diatomite solution. Error bars indicate the standard deviation for four measurements at each data. **c**, Plots of water wicking length L_w on microfibers with varying η versus time. **d**, Diffusion coefficient D versus concentration of diatomite. Values of D are obtained by fitting the plots in (**c**) using the relation $L_w \sim \sqrt{Dt}$.

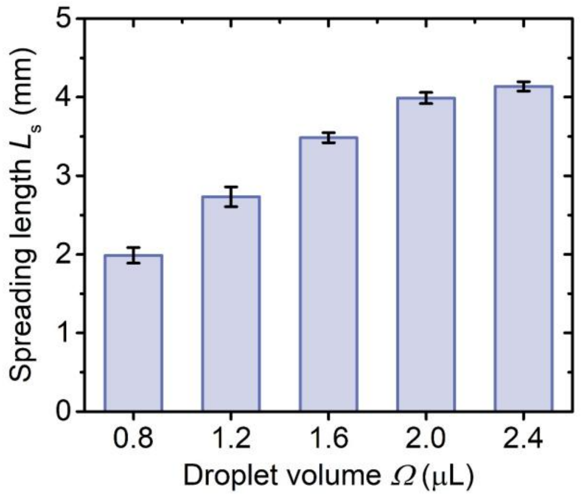

Supplementary Fig. S4: Spreading length versus droplet volume on SEMR. The spacing between microfibers is $s_0 \sim 0.6$ mm. Error bars indicate the standard deviation for three measurements at each data.

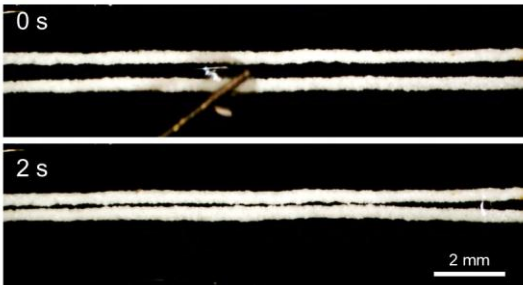

Supplementary Fig. S5: Time-sequence images showing the coalescence of two pure alginate-diatomite microfibers under capillary suction during droplet spreading.

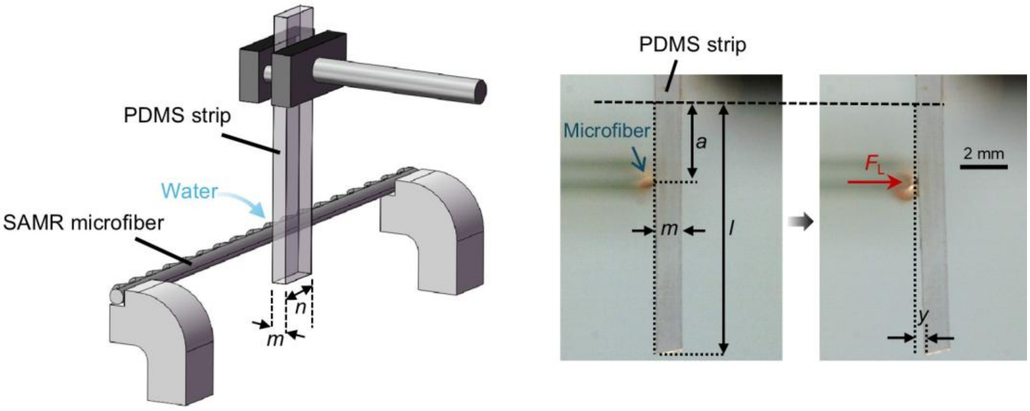

Supplementary Fig. S6: Measurement of force generated by hydroscopic-responsive microfiber deformation.

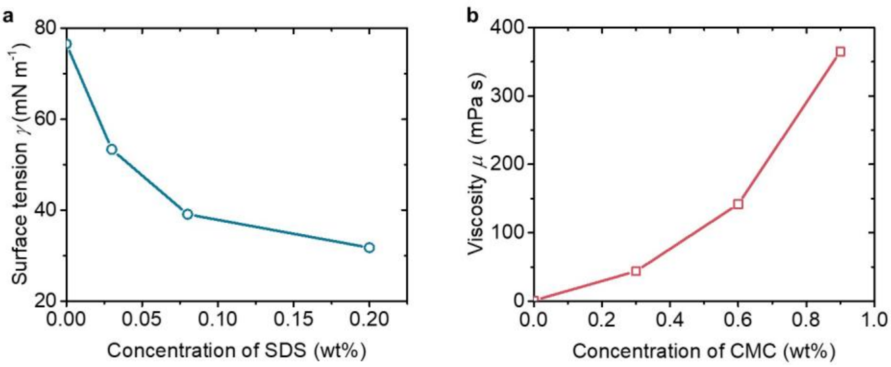

Supplementary Fig. S7: Tuning of fluid property. a, Relationship between surface tension (γ) and concentration of sodium dodecyl sulfate (SDS). As the SDS concentration increases from 0 to 0.2 wt%, γ decreases from ~77 mN m^{-1} to ~32 mN m^{-1}. **b**, Relationship between dynamic viscosity (μ) and concentration of carboxymethyl cellulose (CMC). As the CMC concentration increases from 0 to 0.9 wt%, μ increases from ~1 mPa s to ~365 mPa s.

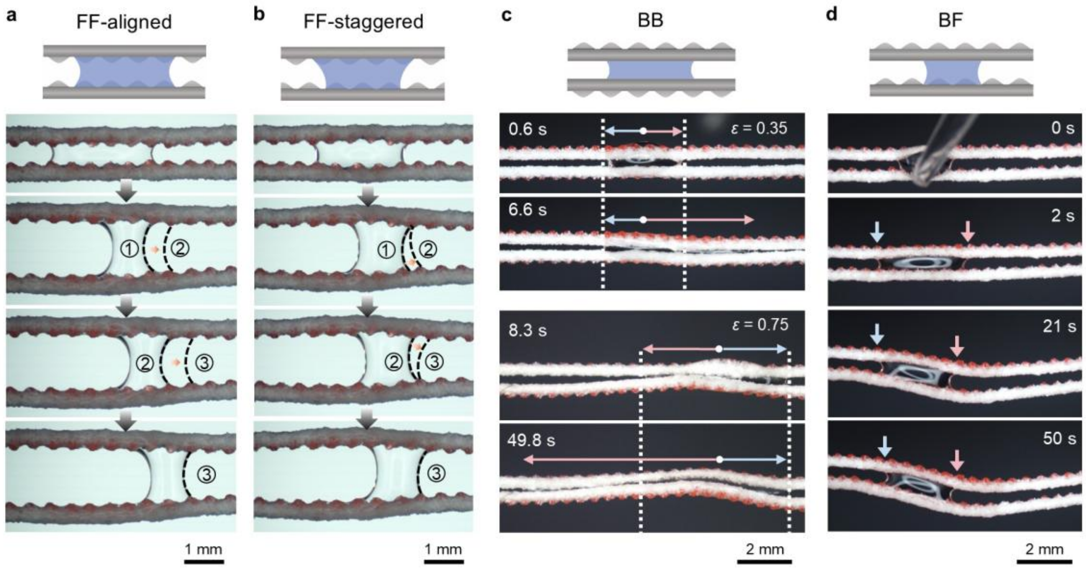

Supplementary Fig. S8: Water droplet transport on microfibers with different configurations. **a**, **b**, Directional water droplet transport on face-to-face (FF) configuration rail with (**a**) aligned and (**b**) staggered surface microstructures, respectively. **c**, Asynchronous water spreading on back-to-back (BB) configuration rail. **d**, A water droplet trapped in back-to-face (BF) configuration rail.

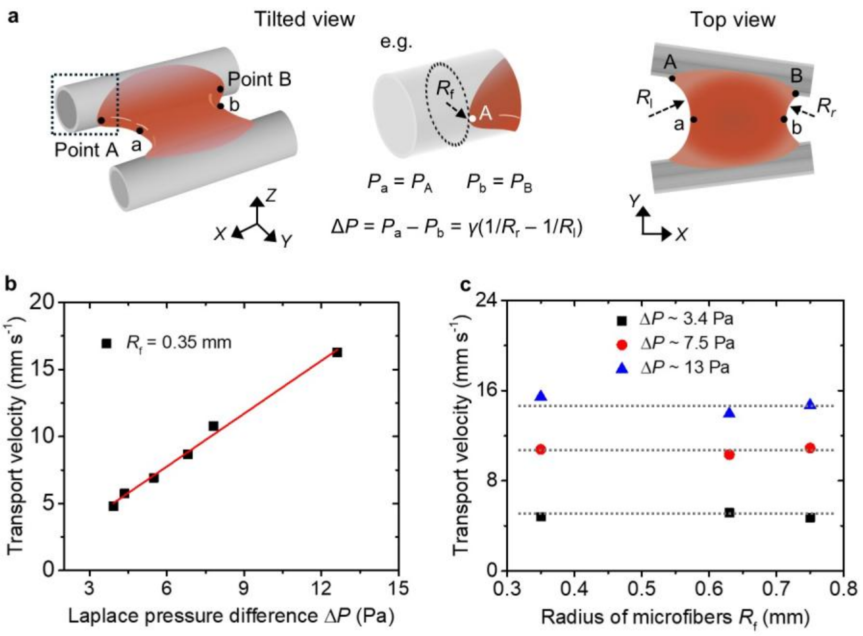

Supplementary Fig. S9: The Laplace pressure difference within the droplet during the transport process. **a**, Schematic of a completely wetting droplet confined between two angled microfibers. **b**, A plot of the transport velocity of ethanol droplets (3 μL) between two non-parallel fibers (fiber radius R_f = 0.35 mm, fiber's wedge angle α = 4.7°) as a function of the Laplace pressure difference ΔP. **c**, Transport velocity of ethanol droplets (3 μL) as a function of the fiber radius R_f under different ΔP.

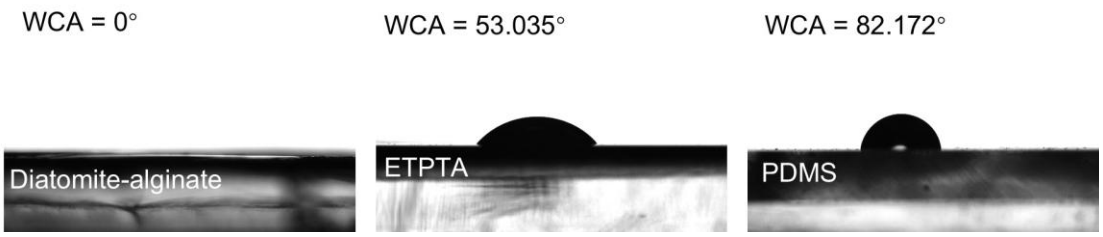

Supplementary Fig. S10: Water contact angles (WCAs) on different substrates. The volume of the water droplet is 1 μL.

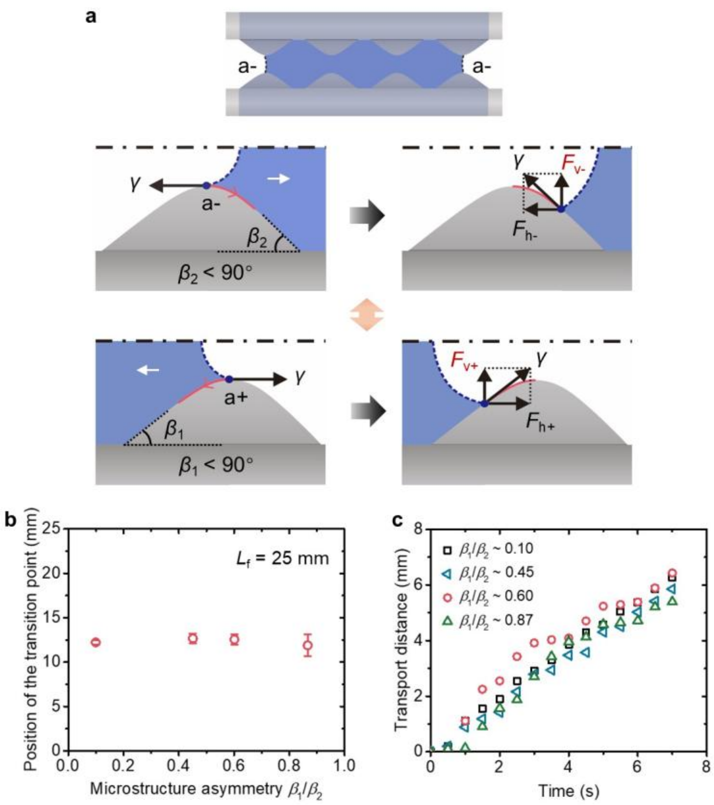

Supplementary Fig. S11: The effect of mound-like microstructure asymmetry on droplet transport performance. a, Schematics depicting the microscopic receding dynamics of droplets on SEMRs with both β_1 and β_2 less than 90°. The mound-like microstructures inhibit contact line retraction on both sides due to the upward-directed force components F_{v+} and F_{v-}. **b**, Variation in the transitional point position for droplet transport direction as a function of microstructure asymmetry ratio β_1/β_2. The microfiber length L_f = 25 mm. Error bars indicate the standard deviation for three measurements at each data. **c**, Transport distance versus time on SEMRs with varied β_1/β_2. The volume of the water droplet is 2 μL.

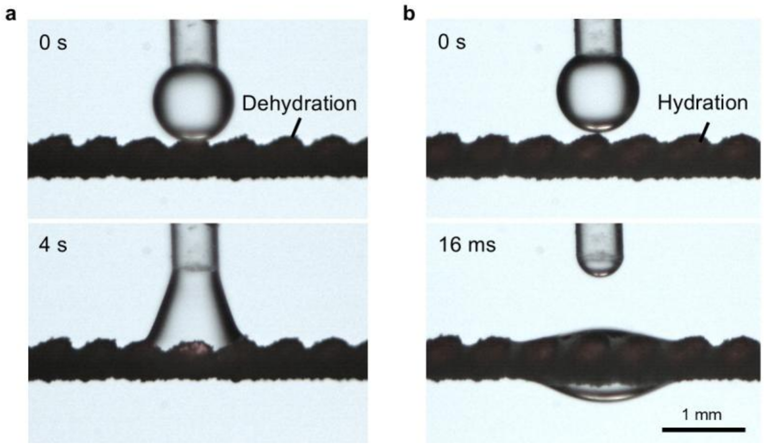

Supplementary Fig. S12: Wetting behavior of water droplets (1 μL) on (**a**) dehydrated and (**b**) hydrated microfibers.

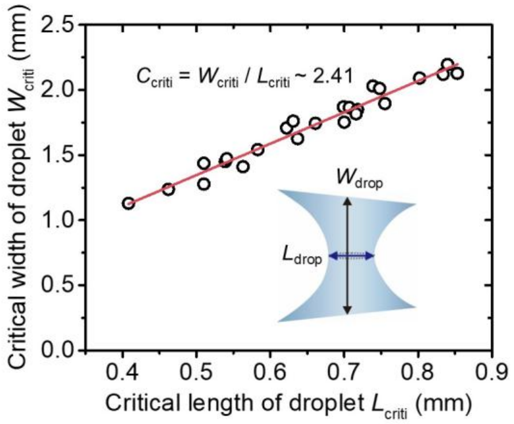

Supplementary Fig. S13: Experimental evaluation of the critical width-to-length aspect ratio C_{criti} at which a droplet breaks.

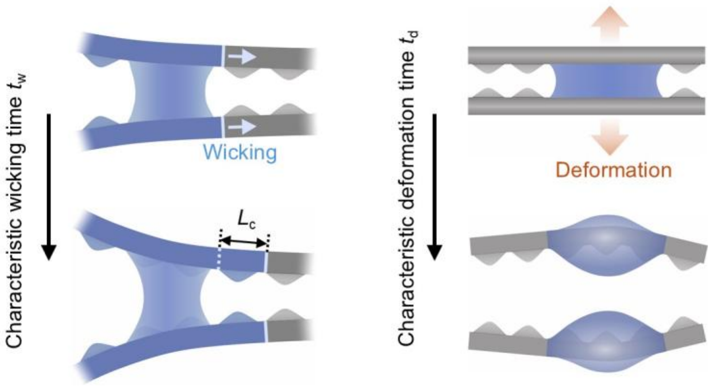

Supplementary Fig. S14: Definitions of characteristic times. The characteristic wicking time (t_w) corresponds to the time required for water to diffuse over a characteristic length (L_c) along the microfiber, where L_c refers to the distance between two adjacent protrusions on the microfiber. The characteristic deformation time (t_d) represents the interval from the onset of microfiber deformation to droplet breakup.

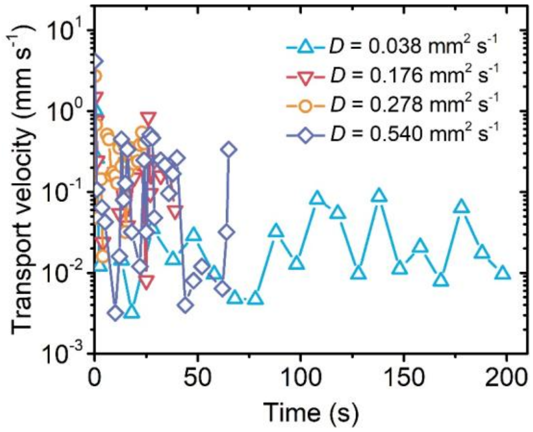

Supplementary Fig. S15: Transport velocity versus time on SEMR with varied diffusion coefficient *D*. The droplet volume is 2 μL.

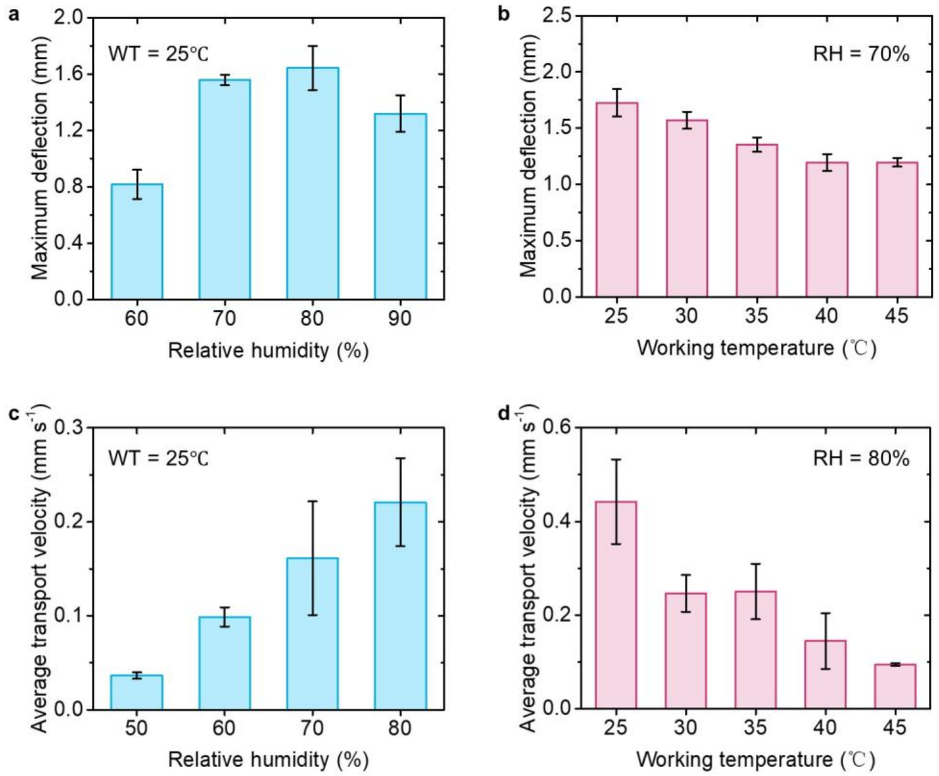

Supplementary Fig. S16: Effects of the environmental conditions on microfiber deformation and droplet transport dynamics. a, **b**, Microfiber deformation under varying humidity levels (**a**) and temperatures (**b**). **c**, **d**, Average transport velocity under varying humidity levels (**c**) and temperatures (**d**). Error bars in (**a**-**d**) indicate the standard deviation for three measurements at each data.

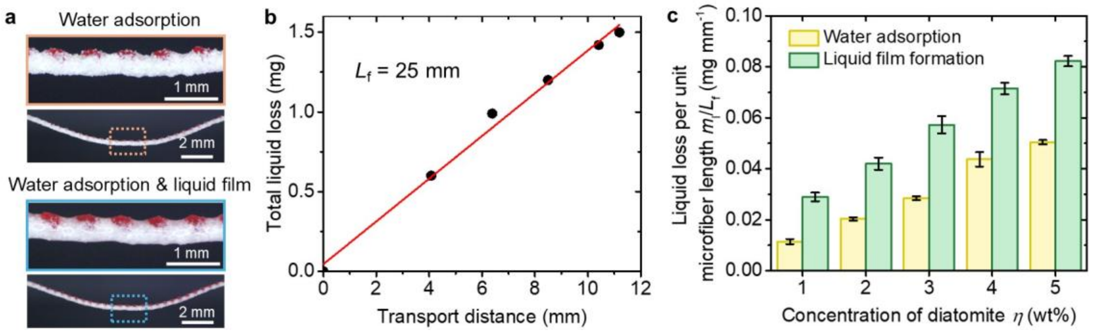

Supplementary Fig. S17: Liquid loss during droplet transport on SEMRs. a, Two mechanisms of liquid loss: (1) water adsorption and (2) liquid film formation. **b**, Total liquid loss as a function of the transport distance. The microfiber length L_f = 25 mm. All droplets have an initial mass of 2.23 mg prior to transport. **c**, Tunable liquid loss per unit length (m_l/L_f) achieved by varying the diatomite concentration η. Error bars indicate the standard deviation for three measurements at each data.

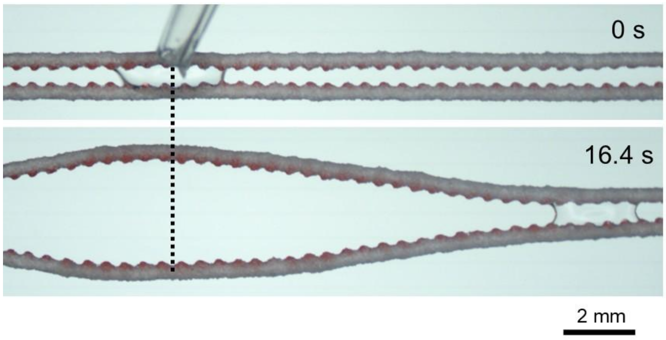

Supplementary Fig. S18: Optical images of a 2 µL water droplet transporting from the deposit position to the right end. The droplet advances ~12 mm in 16.4 s.

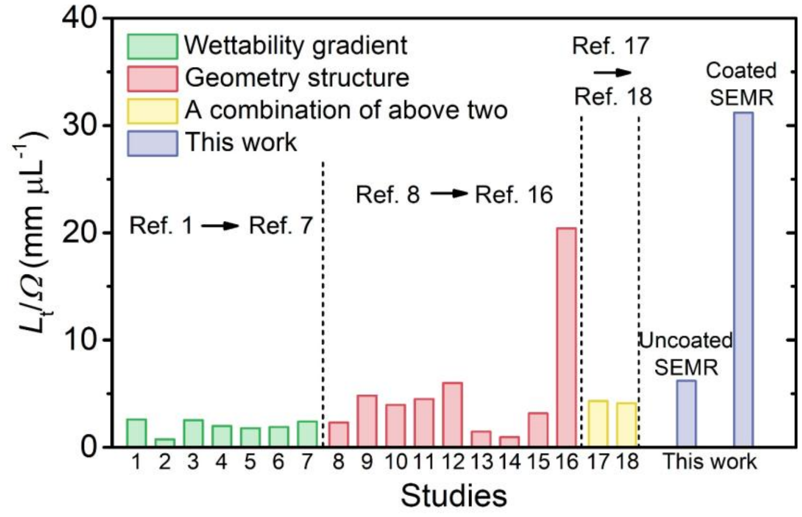

Supplementary Fig. S19: Comparison of transport distance per droplet volume (L_t/Ω) among different surfaces without external energy input.

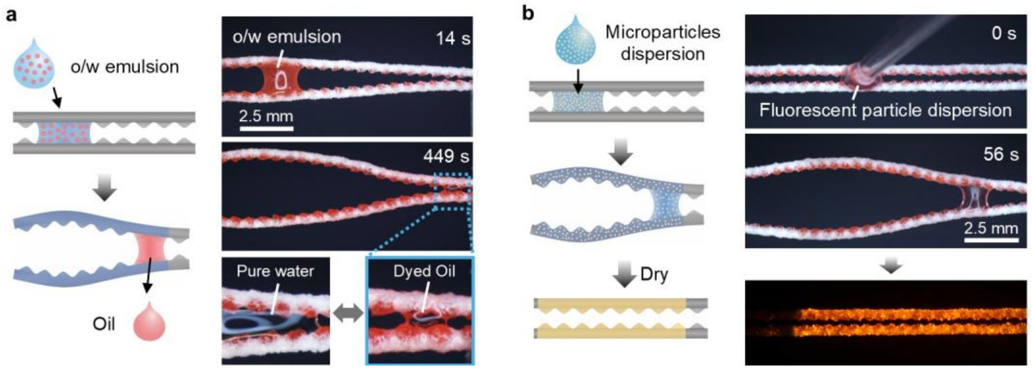

Supplementary Fig. S20: Oil-phase enrichment and microparticle coating enabled by SEMRs. a, Schematic and experimental demonstration of SEMR-mediated oil enrichment through controlled water extraction from oil-in-water emulsions during droplet transport. **b**, Uniform microparticle deposition facilitated by the residual liquid film. The fluorescence photograph (bottom) confirms homogeneous microparticle distribution on SEMR surfaces after droplet transport.

Supplementary References

1 Li, J. *et al.* Oil droplet self-transportation on oleophobic surfaces. *Sci. Adv.* **2**, e1600148 (2016).

2 Sun, C., Zhao, X. W., Han, Y. H., & Gu, Z. Z. Control of water droplet motion by alteration of roughness gradient on silicon wafer by laser surface treatment. *Thin Solid Films* **516**, 4059-4063 (2008).

3 Bliznyuk, O., Jansen, H. P., Kooij, E. S., Zandvliet, H. J., & Poelsema, B. Smart design of stripe-patterned gradient surfaces to control droplet motion. *Langmuir* **27**, 11238-11245 (2011).

4 Zhu, X., Wang, H., Liao, Q., Ding, Y. D., & Gu, Y. B. Experiments and analysis on self-motion behaviors of liquid droplets on gradient surfaces. *Exp. Therm. Fluid. Sci.* **33**, 947-954 (2009).

5 Chaudhury, M. K., & Whitesides, G. M. How to make water run uphill. *Science* **256**, 1539-1541 (1992).

6 Ito, Y. *et al.* The movement of a water droplet on a gradient surface prepared by photodegradation. *Langmuir* **23**, 1845-1850 (2007).

7 Launay, G. *et al.* Self-propelled droplet transport on shaped-liquid surfaces. *Sci. Rep.* **10**, 14987 (2020).

8 Jang, H., Lee, H. S., Lee, K. S., & Kim, D. R. Facile fabrication of superomniphobic polymer hierarchical structures for directional droplet movement. *ACS Appl. Mater. Interfaces* **9**, 9213–9220 (2017).

9 Zhang, X. *et al.* Lossless and directional transport of droplets on multi-bioinspired superwetting V-shape rails. *Adv. Funct. Mater.* **33**, 2212217 (2023).

10 Park, K. C. *et al.* Condensation on slippery asymmetric bumps. *Nature* **531**, 78-82 (2016).

11 Lv, C. *et al.* Substrate curvature gradient drives rapid droplet motion. *Phys. Rev. Lett.* **113**, 026101 (2014).

12 Khoo, H. S., & Tseng, F. G. Spontaneous high-speed transport of subnanoliter water droplet on gradient nanotextured surfaces. *Appl. Phys. Lett.* **95**, 063108 (2009).

13 Zhuang, K., Lu, Y., Wang, X., & Yang, X. Architecture-driven fast droplet transport without mass loss. *Langmuir* **37**, 12519-12528 (2021).

14 Bradley, A. T., Box, F., Hewitt, I. J., & Vella, D. Wettability-independent droplet transport by Bendotaxis. *Phys. Rev. Lett.* **122**, 074503 (2019).

15 Chen, Y. *et al.* Bioinspired superwettable microspine chips with directional droplet transportation for biosensing. *ACS nano* **14**, 4654-4661 (2020).

16 Jiang, J. *et al.* Directional pumping of water and oil microdroplets on slippery surface. *Proc. Natl. Acad. Sci. U. S. A.* **116**, 2482-2487 (2019).

17 Li, Y. *et al.* Enhanced movement of two-component droplets on a wedge-shaped Ag/Cu surface by a wettability gradient. *ACS Appl. Mater. Interfaces* **13**, 15857-15865 (2021).

18 Liu, Q. *et al.* Achieving ultralong directional liquid transportation spontaneously with a high velocity. *J. Mater. Chem. A* **11**, 10164-10173 (2023).
