# Supplementary material for: Spontaneous droplet transport on shape-evolving microfiber rails
Source: Nat Commun. 2025 Dec 4;16:10894. doi: 10.1038/s41467-025-65884-9 (PMC12678407; doi:10.1038/s41467-025-65884-9)
Supplement: Supplementary file 2 — Description of Additional Supplementary Files [file 41467_2025_65884_MOESM2_ESM.pdf]

Description of Additional Supplementary Files

File Name: Supplementary Video 1
Description: Microfiber fabrication. This video illustrates the shape transformations of internal oil droplets shortly after the aqueous alginate-diatomite jet encounters the $CaCl_2$ solution. These changes are induced by asymmetric shear forces acting on both sides of the droplet. The external flow rate is 1.4 mL h^{-1}, and the internal flow rate is 0.09 mL h^{-1}.

File Name: Supplementary Video 2
Description: Selective directional transport of droplets on SEMR featuring symmetric microstructures. This video showcases droplets placed on SEMR with symmetric microstructures, which choose different directional transport paths based on their initial positions, preferentially moving toward the nearest fixed ends of the microfiber rail.

File Name: Supplementary Video 3
Description: The directional transport of a droplet on SEMR featuring asymmetric microstructures. The video showcases a droplet placed on SEMR with asymmetric microstructures, moving directionally along the inclined ratchet on the surface.

File Name: Supplementary Video 4
Description: The droplet retraction governed by the asymmetric microstructures of SEMR. This video illustrates the droplets retracting only along the tilted direction of the ratchet on the SEMR with asymmetric microstructures.

File Name: Supplementary Video 5
Description: The directional transport of a water droplet on SEMR. This video presents a 2 μL droplet placed on SEMR, moving directionally over a distance of ~12 mm.

File Name: Supplementary Video 6
Description: Spontaneous coalescence of droplets on SEMR. This video illustrates the coalescence of two droplets placed on SEMR. Initially, both droplets move at similar speeds. Subsequently, the trailing droplet experiences a sudden increase in speed, quickly colliding with and merging into the leading droplet. The coalesced droplet then continues to advance along the rail.

File Name: Supplementary Video 7
Description: The directional transport of oil droplets on SEMR. Oil droplets placed on SEMR only undergo a spreading process and finally remain stationary. By adding water droplets, a gradient is induced on SEMR to drive the directional transport of the oil droplets with varied viscosities.

File Name: Supplementary Video 8
Description: The directional transport of a solid sphere on SEMR. This video demonstrates the use of a water droplet as a transport carrier to achieve directional movement of a solid sphere.

File Name: Supplementary Video 9
Description: Intelligent liquid circuit. By altering the position of droplets added to SEMR, the direction of droplet transport can be controlled, thereby lighting up different

circuits.

File Name: Supplementary Video 10
Description: Component detection based on SEMR. A 2 μL citric-acid droplet traverses a region on SEMR loaded with cresol red, changing the area from purple to yellow and indicating the presence of acidic substances in the droplet.
